# Chronic wasting disease prions in mule deer interdigital glands

**DOI:** 10.1371/journal.pone.0275375

**Published:** 2022-10-03

**Authors:** Anthony Ness, Doris Zeng, Alsu Kuznetsova, Alicia Otero, Chiye Kim, Kelsey Saboraki, Susan Lingle, Margo Pybus, Judd Aiken, Sabine Gilch, Debbie McKenzie

**Affiliations:** 1 Department of Biological Sciences, University of Alberta, Edmonton, Alberta, Canada; 2 Centre for Prions and Protein Folding Diseases, Edmonton, Alberta, Canada; 3 Department of Comparative Biology and Experimental Medicine, University of Calgary, Calgary, Alberta, Canada; 4 Hotchkiss Brain Institute, University of Calgary, Calgary, Canada; 5 Department of Agricultural, Food and Nutritional Sciences, University of Alberta, Edmonton, Alberta, Canada; 6 Department of Biology, University of Winnipeg, Winnipeg, Manitoba, Canada; 7 Alberta Environment and Parks, Edmonton, Alberta, Canada; The University of Texas Health Science Center at Houston, UNITED STATES

## Abstract

Chronic wasting disease (CWD) is a geographically expanding, fatal neurodegenerative disease in cervids. The disease can be transmitted directly (animal-animal) or indirectly via infectious prions shed into the environment. The precise mechanisms of indirect CWD transmission are unclear but known sources of the infectious prions that contaminate the environment include saliva, urine and feces. We have previously identified PrP^C^ expression in deer interdigital glands, sac-like exocrine structures located between the digits of the hooves. In this study, we assayed for CWD prions within the interdigital glands of CWD infected deer to determine if they could serve as a source of prion shedding and potentially contribute to CWD transmission. Immunohistochemical analysis of interdigital glands from a CWD-infected female mule deer identified disease-associated PrP^CWD^ within clusters of infiltrating leukocytes adjacent to sudoriferous and sebaceous glands, and within the acrosyringeal epidermis of a sudoriferous gland tubule. Proteinase K-resistant PrP^CWD^ material was amplified by serial protein misfolding cyclic amplification (sPMCA) from soil retrieved from between the hoof digits of a clinically affected mule deer. Blinded testing of interdigital glands from 11 mule deer by real-time quake-induced conversion (RT-QuIC) accurately identified CWD-infected animals. The data described suggests that interdigital glands may play a role in the dissemination of CWD prions into the environment, warranting future investigation.

## Introduction

Chronic wasting disease (CWD) is a contagious, fatal transmissible spongiform encephalopathy of cervids. The pathological agent of CWD (PrP^CWD^) is a misfolded isoform of the cellular prion protein (PrP^C^) that propagates by a template misfolding-like mechanism [1–3]. The environment of CWD-endemic areas is contaminated by clinically and subclinically affected animals through feces, carcasses, and body fluids including urine and saliva [4–12]. Prions shed into the environment can remain infectious for years to decades—contributing to horizontal disease transmission [13–16]. Shed prions bind to soil, vegetation, and other fomites that provide reservoirs for naïve deer exposure [12, 17–23].

Deer possess a number of integumentary glands that have been hypothesized to be involved in CWD transmission [24–26]. Many cervids, including mule deer (*Odocoileus hemionus*) and white-tailed deer (*Odocoileus virginianus*), but not elk (*Cervus canadensis*), possess interdigital glands—anteriorly-facing pocket structures between the two first phalangeal bones—in the fore and hind feet [27–29]. Interdigital glands secrete volatile compounds that are believed to create scent trails [27, 28, 30–32]. We previously reported the distribution of PrP^C^ in mule deer and white-tailed deer integumentary glands [26]. PrP^C^ expression within the interdigital glands was observed within the sebaceous glands, sudoriferous glands, portions of epidermis, hair root sheaths, and infiltrating immune cells. A parallel survey for the presence of disease-associated PrP^CWD^ in the tissues was performed. We identified PrP^CWD^ within a hind interdigital gland of a CWD-infected female mule deer, leading to a further investigation of this cutaneous gland. Our subsequent analysis of blinded interdigital gland samples from 11 mule deer resulted in the correct identification of infected animals. We describe the findings of PrP^CWD^ being found within the interdigital glands of mule deer and discuss the associated implications.

## Materials and methods

### Tissue collection

**Fig 1:**
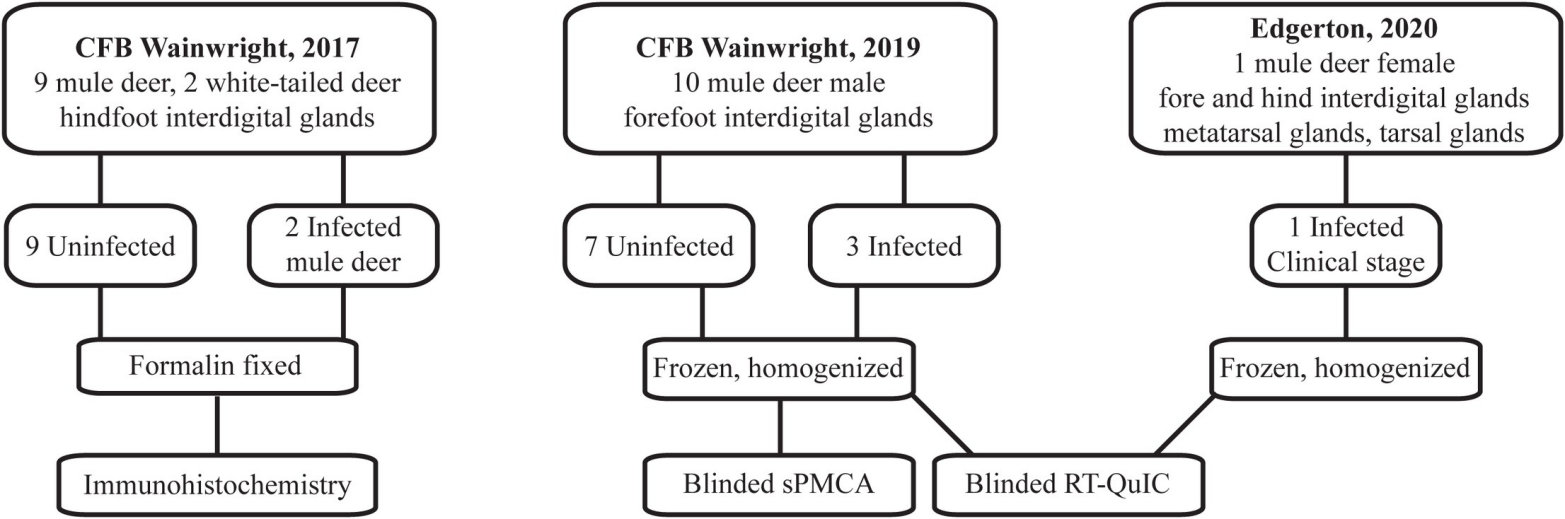
Conceptual diagram of sample origin and use.

All deer samples were obtained from animals harvested by hunters or found dead. Institutional animal care and use approval was not required for the tissues used this study. Tissue samples used in this study were collected at three different times (Fig 1). Hunter harvested deer samples from 2017 and 2019 used in this study were collected from Albertan Canadian Forces Base (CFB) Wainwright, Alberta. Tissues collected for the 2017 PrP^CWD^ survey included the apical portion of the vomeronasal organ, the parotid gland, and 6 integumentary glands—forehead, preorbital, vestibular nasal, tarsal, metatarsal, and hind interdigital glands. This study focuses on the interdigital glands. Samples were frozen for later biochemical analysis or formalin-fixed for histology. Investigation into the distribution of PrP^CWD^ in the tissues described was limited by random selection of deer to be sampled. CWD status of individual deer was unknown during tissue collection. The CWD status (positive or negative) of sampled deer was later determined by the Alberta Ministry of Agriculture and Forestry using the Bio-Rad TeSeE ELISA on retropharyngeal lymph nodes and/or obex samples [33]. Consequently, for the broad gland collection of 41 deer in 2017 (including mule deer and white-tailed deer), only 2 were positive for CWD—both mule deer. The two hunter-harvested adult mule deer (1 of each sex) were CWD-positive in the retropharyngeal lymph nodes and obex (specific obex scoring unavailable). Hunter-reported fat levels (indicative of possible clinical-stage wasting) of the two infected deer was listed as normal.

Additional frozen forelegs were obtained from 10 male mule deer harvested by hunters within CFB Wainwright in the autumn of 2019. Male mule deer have the highest prevalence of CWD in Alberta relative to females and white-tailed deer [33]. During the 2019 harvest, male mule deer at CFB Wainwright (wildlife management units 728 and 730) had an estimated prevalence of 11–30% [34]. Interdigital glands were extracted from the hooves as follows. The forelegs were placed in plastic bags to prevent wetting while being thawed by warm water. Local pelage was trimmed with straight-blade stainless steel operating scissors prior to dissection. Scissors were decontaminated with 2M NaOH submersion for 24 hours between leg sets. Each interdigital gland was excised with a single-use disposable scalpel to prevent cross contamination. Once the interdigital gland was exposed (S1A Fig in S1 File), the fundus of each gland sac was excised with a disposable razor (S1B Fig in S1 File) and frozen for later homogenization.

Additional Albertan tissue samples were collected from a collared yearling female mule deer with signs of wasting that was found dead in eastern Alberta during the October of 2020. The animal is presumed to have died of clinical CWD. The animal was tested by the Alberta Ministry of Agriculture and Forestry as described above and was found to be CWD-positive. All four legs (including the hind tarsal and metatarsal glands) were removed and frozen. The interdigital gland from each leg and both tarsal and metatarsal glands from the hind legs were extracted and individually homogenized.

### *PRNP* gene amplification and sequencing

DNA from all CWD-infected animals was extracted and sequenced to identify any *PRNP* genotypes. Genomic DNA was isolated from deer tissues by methanol precipitation. The cervid *PRNP* gene was amplified by a primer set targeting the coding region of the mature mule deer prion protein using GoTaq® Long PCR Master Mix (Promega Corporation, USA). To avoid amplification of the mule deer prion protein pseudogene (GenBank accession no. AY371694) or white-tailed deer prion protein pseudogene (GenBank accession no. AY425673), primers (DeerPCR1F and DeerPCR7F) were specifically designed for the sequence region with high variation between the white-tailed deer and mule deer prion protein pseudogenes and the functional mule deer *PRNP* gene (GenBank accession no. AY228473). The forward primer, DeerPCR1F (5’-ACCTACAATTACTTTCGTGAGATGT-3’), overlaps intron 2 and the reverse primer, DeerPCR1R (5’-CAAGAAATGAGACACCACCACTA-3’), was located 1059 bp downstream of the forward primer (GenBank accession no. AY228473). PCR thermocycling conditions were 95°C for 3 min for initial denaturation followed by 35 cycles of 95°C for 30 s, 62°C for 30 s, and 65°C for 2 min, and the final extension at 72°C for 10 min. PCR fragments were sequenced by Sanger DNA sequencing at the University of Alberta Molecular Biology Facility using the forward primer DeerPCR7F (5’-CTGATGCCACTGCTATGCAGTCAT-3’) and the reverse primer DeerPCR1R with BigDye® sequencing reagents (ThermoFisher Scientific, United States).

### Histological and immunohistochemical detection of PrP^CWD^ in gland tissues

Formalin fixed glands were trimmed and embedded in paraffin. 4μm thick sections were cut using a microtome and mounted on glass slides. Sections were then stained using the hematoxylin and eosin (H&E) protocol to identify the histological structures of the glands. Immunohistochemical labeling for the detection of PrP^CWD^ in glandular tissues required modifications to common methods used for brain tissues previously described to prevent the loss of fine structures and leukocytic infiltrates [35]. Tissue sections were incubated at 65°C overnight, deparaffinized and dehydrated by immersion in xylene and decreasing concentrations of ethanol (100%, 95% and 70%). Epitope retrieval was performed by hydrated autoclaving at 121°C in deionized water for 10 minutes, incubated with 98% formic acid for 10 minutes, digested with proteinase K (4μg/mL) (Invitrogen, USA) at 37°C for 15 minutes, and incubation with 4M guanidine thiocyanate for 2 hours. Endogenous peroxidase was blocked using a 3% hydrogen peroxide solution for 12 minutes. Sections were exposed to 5% goat serum for 1 hour to block non-specific sites followed by 15 minutes of blocking with avidin and biotin (Vector Laboratories, USA) respectively. Immunodetection was completed by incubating the samples with the monoclonal antibody BAR224 (1:2,000; Cayman Chemical, USA) overnight at 4°C followed by 1 hour of incubation with an anti-mouse horseradish peroxidase (HRP) secondary antibody (1:250; Immun-Star). AEC (3-Amino-9-ethylcarbazole) (Vector Laboratories, USA) was used as the HRP chromogen substrate. After counterstaining with hematoxylin, sections were mounted with DPX mounting medium (MilliporeSigma, USA). Control slides in which incubation with the primary antibody was omitted were used for specificity controls of the technique. Slides were scanned with a NanoZoomer 2.ORS (Hamamatsu Photonics K.K., Japan) and the images analyzed with the manufacturer’s NDP.view2 software.

### Tissue homogenization

Glandular tissue was processed as detailed previously [26]. The tissues were weighed and minced to create 10% (w/v) homogenates in RIPA lysis buffer (50mM Tris, 150mM NaCl, 1% IGEPAL CA-630, 0.25% deoxycholate, 1mM EDTA, pH 7.4) supplemented with cOmplete™ EDTA-Free Protease Inhibitor Cocktail (Roche Diagnostics GmbH, Switzerland). Samples were mechanically homogenized in a Bead Ruptor 24 (Omni International, USA) ceramic bead mill homogenizer in the presence of a cold air flow supplied by an OMNI BR-Cryo cooling unit (Omni International, USA) to minimize heat-denaturation. Tissues were subjected to 25 minutes of high-energy milling using cycles of 10 second milling intervals followed by 15 seconds of cooling. Homogenate supernatants were collected following a brief centrifugation to yield clarified 10% gland homogenates for biochemical analysis.

### Serial protein misfolding cyclic amplification and western blot analysis

Interdigital clarified gland homogenates blinded for CWD status were analyzed by sPMCA. PMCA substrate preparation was prepared from transgenic mice expressing elk 132MM prion protein (tgElk) [36]. Mice were perfused, after euthanasia by isoflurane inhalation, using phosphate buffered saline (PBS) (130mM NaCl, 7mM Na_2_HPO_4_·7H_2_O, 3mM NaH_2_PO_4_·1H_2_O, pH 7.4) with 5mM EDTA (University of Alberta Animal Use Protocol 914). Brains were extracted and immediately frozen at -80°C. The brain substrate (10% w/v brain homogenate) was prepared using a Dounce tissue grinder, homogenizing the brain tissue in chilled PMCA conversion buffer (PBS, 150mM NaCl, 1% Triton X-100, 4mM EDTA) with cOmplete™ EDTA-Free Protease Inhibitor Cocktail (Roche Diagnostics GmbH, Switzerland) and clarified by centrifugation at 800g for 5 min. Supernatant aliquots (90μL each) were stored in 0.2mL PCR tubes (Corning, USA) at -80°C. In vitro amplification of PrP^CWD^ present in gland homogenates was performed using serial protein misfolding cyclic amplification (sPMCA) similarly to that described previously [37–40]. Each PMCA reaction included three 3/32″ PTFE beads (McMaster-Carr, USA) to increase the efficiency of prion amplification. Substrates seeded with 10μL of samples were placed on the plate holder of a S-4000 Misonix sonicator (QSonica, USA) and were subjected to 24 hour rounds of serial PMCA consisting of incubation cycles of 15 min at 37°C followed by sonication pulses of 30s at 60% power.

Reaction products sPMCA rounds were examined for evidence of PrP^CWD^ seeding by western blot. Samples of sPMCA round products were proteolytically digested using 50μg/ml of proteinase K (PK) for 1 h at 37°C with agitation. Digestion was terminated by the addition of 2x Laemmli sample buffer (150mM Tris-HCl (pH 6.8), 0.5% bromophenol blue, 25% (v/v) glycerol, 5% (w/v) SDS, 12.5% (v/v) 2-mercaptoethanol) and boiling at 100°C for 10min. Samples were analyzed by western blot using Invitrogen NuPAGE™ 12% Bis-Tris protein gels (ThermoFisher Scientific Inc., USA) separated in MOPS buffer. Proteins were transferred onto PVDF membranes, followed by immunodetection of PrP^CWD^ with mouse anti-PrP monoclonal antibody SHA31 (1:10,000; Cayman Chemical, USA). Blots were developed using the AttoPhos AP Fluorescent Substrate System (1:10,000; Promega Corporation, USA). Secondary goat anti-mouse IgG (H+L) AP Conjugate (Promega Corporation, USA) detection antibody was enzymatically developed with AttoPhos® AP Fluorescent Substrate System (Promega Corporation, USA). Fluorescent imaging of the membranes was performed using an ImageQuant LAS 4000 (GE Life Sciences, USA) system.

### Real-time quaking-induced conversion (RT-QuIC)

RT-QuIC using recombinant mouse PrP substrate was performed as previously described [41, 42]. Briefly, RT-QuIC reactions were set up in assay buffer containing 20 mM sodium phosphate (pH 6.9), 300 mM NaCl, 1 mM EDTA, 10 μM thioflavin T (ThT), and 0.1 mg/ml full-length mouse recombinant PrP (amino acids 23–230) substrate. Aliquots of 98μL were added to the wells of a 96 well optical bottom plate (Nalge Nunc International, USA). Quadruplicate reactions were seeded with 2μl of sample. Clarified 10% (w/v) interdigital, metatarsal, and tarsal gland homogenates were blinded for CWD status and assayed for the presence of prion infectivity by RT-QuIC. Mouse recombinant PrP, in our hands, efficiently amplifies CWD prions with minimal spontaneous conversion (we find higher levels of spontaneous conversion with both bank vole and truncated hamster PrP). Uninfected mule deer and CWD-infected reindeer brain homogenates were used for negative and positive controls. Gland and control brain homogenates were assayed at dilutions of 1:20 (0.5% final concentration), and 1:200 (0.05% final concentration).

The plate was sealed with Nunc Amplification Tape (Nalge Nunc International, USA) and placed in a FLUOstar Omega fluorescence plate reader (BMG LABTECH GmbH, Germany) that was pre-heated to 42˚C. The RT-QuIC assay was run for a total of 50 hours with cycles of 1 minute of double orbital shaking (700 rpm) incubation and 1 minute of resting throughout the incubation. ThT fluorescence signals (450nm excitation, 480nm emission) of each well were recorded every 15 minutes. The positive sample threshold was calculated using the average ThT fluorescence signals of the negative control +5 standard deviations. Values were plotted as the average of quadruplicate reactions by using GraphPad Prism software (GraphPad Software, USA).

### Soil collection and prion detection

A soil sample was obtained from the clinically affected female mule deer. The soil was lodged between the two hoof digits in the vicinity of the interdigital gland. The hoof soil was not characterized due to limited sample size, but the deer legs were collected near Edgerton, Alberta where dark brown and black chernozemic soils are predominant [43]. A comparable negative control (orthic black chernozem, humic horizon Ah) soil sample was sourced from the Leduc region of Alberta in 2010 when the area was CWD-free. The control soil sample has been previous described [44]. Bound PrP^CWD^ was extracted from soil samples as follows. Soil samples were mixed with 5x Laemmli buffer (300mM tris base, 50% (v/v) glycerol, 10% (w/v) sodium dodecyl sulfate, 25% (v/v) 2-mercaptoethanol) in ratio 1:1 and heated for 10 minutes at 100°C. Samples were briefly centrifuged, then 10μL of sample supernatant were used to seed tgElk PMCA substrate following brief centrifugation. Subsequent sPMCA was performed as described above.

## Results

### Detection of interdigital PrP^CWD^ by immunohistochemistry

**Fig 2:**
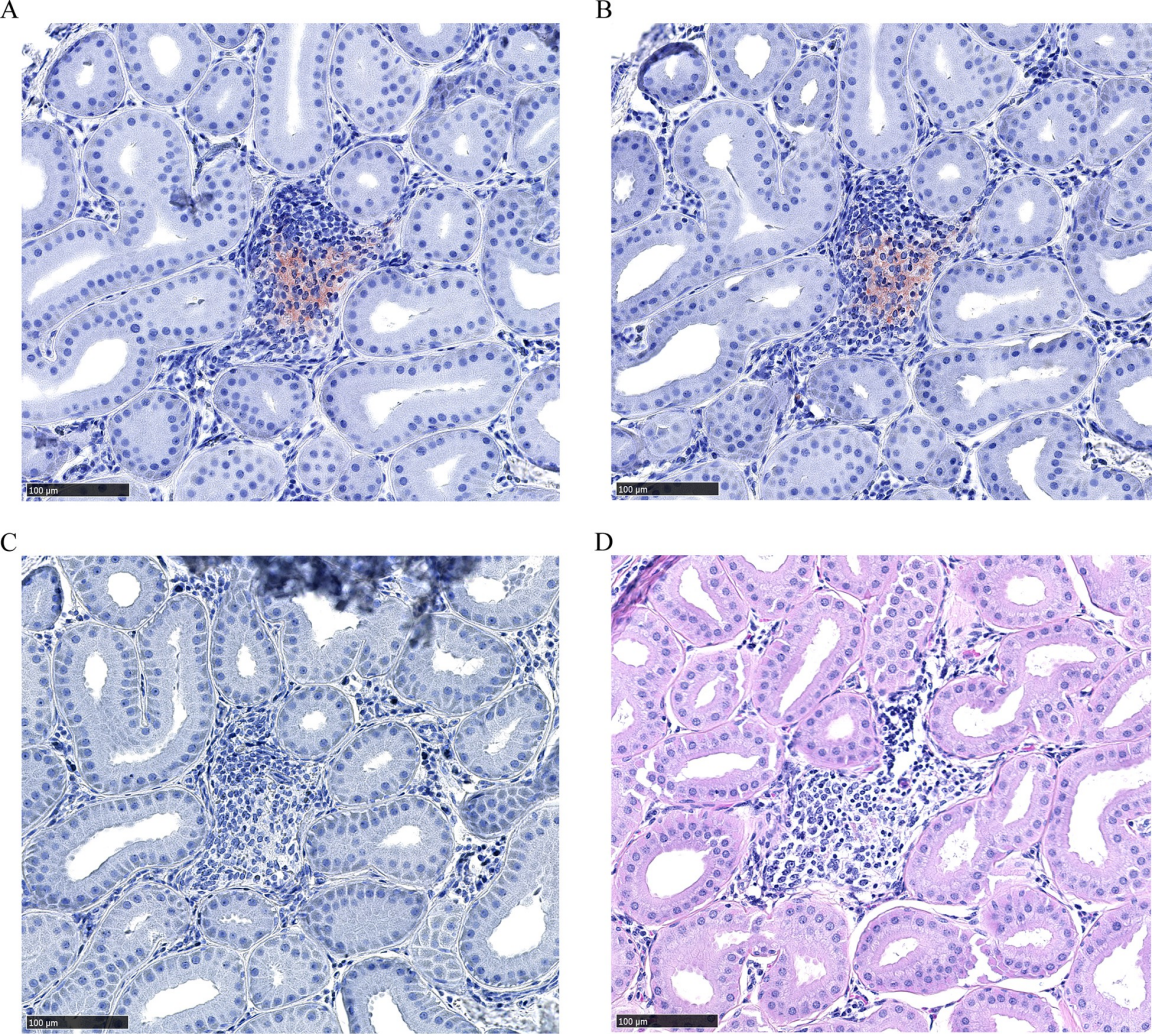
PrP^CWD^ immunolabeling between sudoriferous glands of a female mule deer hind interdigital gland. (A-B) Adjacent sections of immune cell infiltrates between sudoriferous glands with PrP^CWD^ immunolabeling (red). PrP^CWD^ was immunolabeled with anti-PrP BAR224 (1:2,000). (C) Negative control section without primary antibody. (D) Hematoxylin and eosin staining.

**Fig 3:**
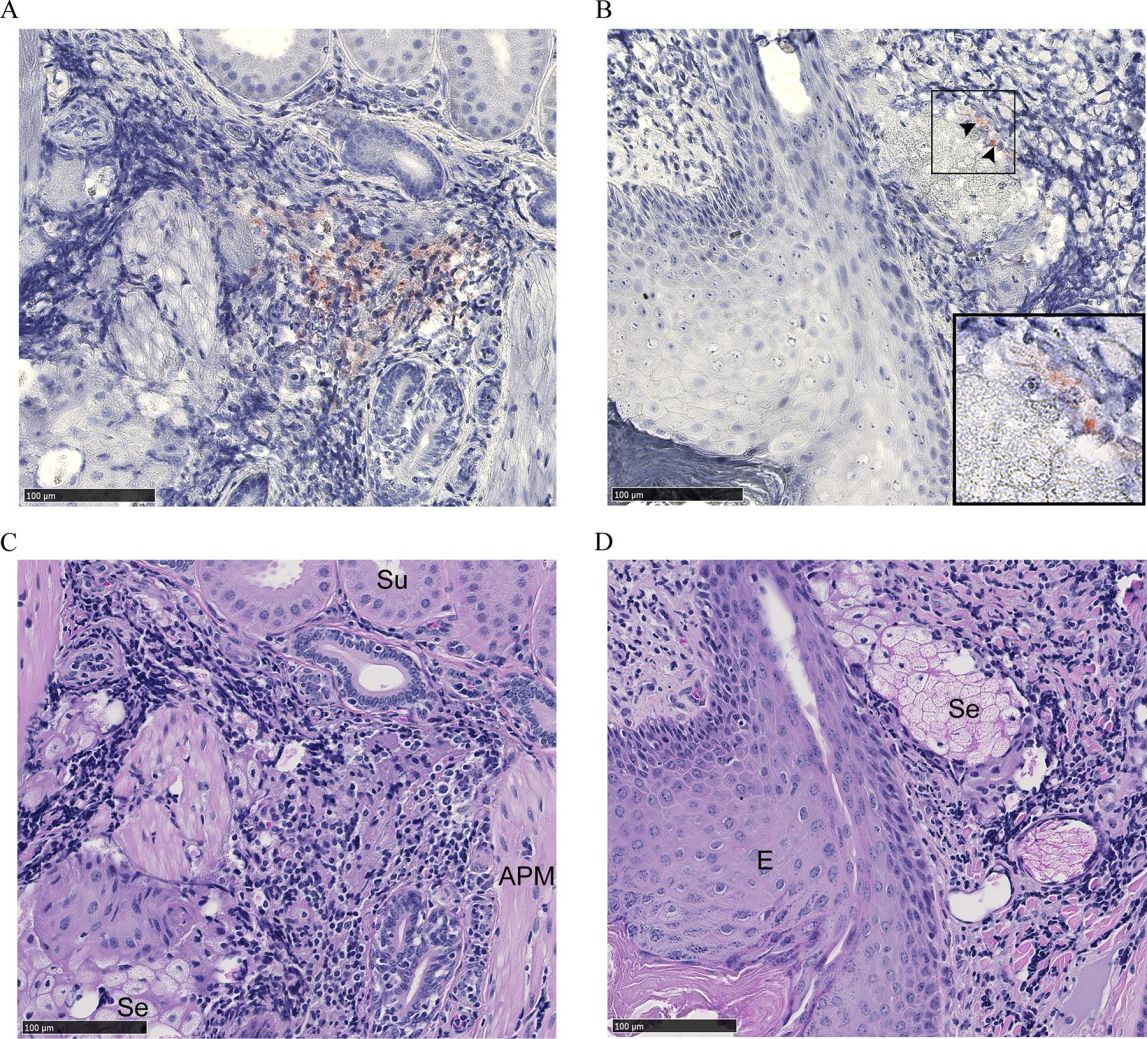
PrP^CWD^ immunolabeling adjacent to sudoriferous and sebaceous glands of a female mule deer hind interdigital gland. (A) Immune cell infiltrates between sebaceous and sudoriferous glands with PrP^CWD^ immunolabeling (red). (B) Immune cell infiltrates near the epidermis with PrP^CWD^ immunolabeling (arrows) adjacent to a sebaceous glandular element. Inset shows PrP^CWD^ with increased magnification. PrP^CWD^ was immunolabeled with anti-PrP BAR224 (1:2,000). (C-D). Adjacent section hematoxylin and eosin staining. Abbreviations: APM, arrector pili muscle; E, epidermis; Se, sebaceous gland; Su, sudoriferous gland.

**Fig 4:**
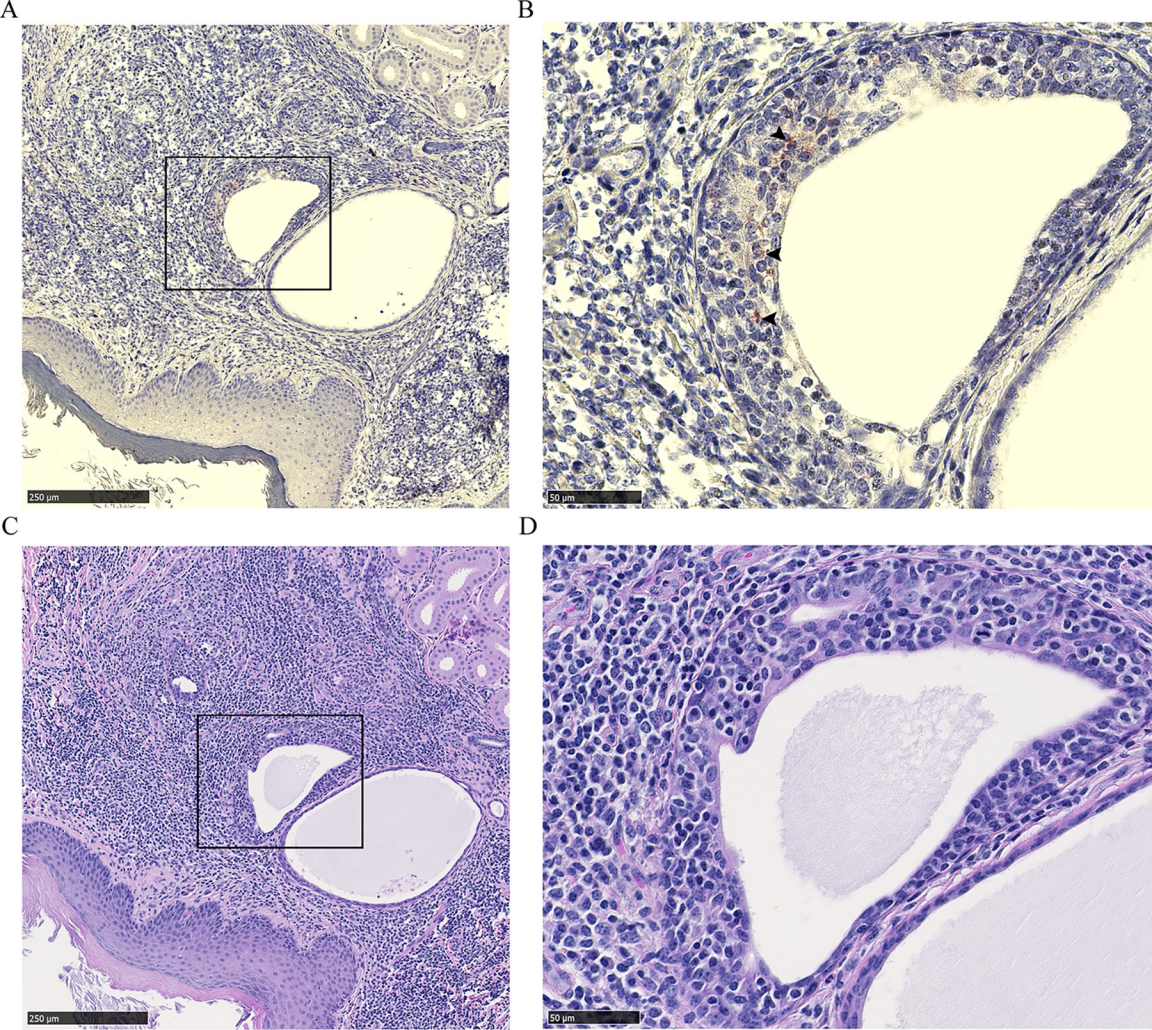
PrP^CWD^ immunolabeling within the acrosyringeal epidermis of a female mule deer hind interdigital gland. (A) Immune cell infiltrates between epidermis and sudoriferous glands. (B) Increased magnification of the inset showing PrP^CWD^ immunolabeling (arrows) within the acrosyringeal epidermis of a dilated sudoriferous tubule. PrP^CWD^ was immunolabeled (red) with anti-PrP BAR224 (1:2,000). (C-D) Adjacent section hematoxylin and eosin staining.

**Fig 5:**
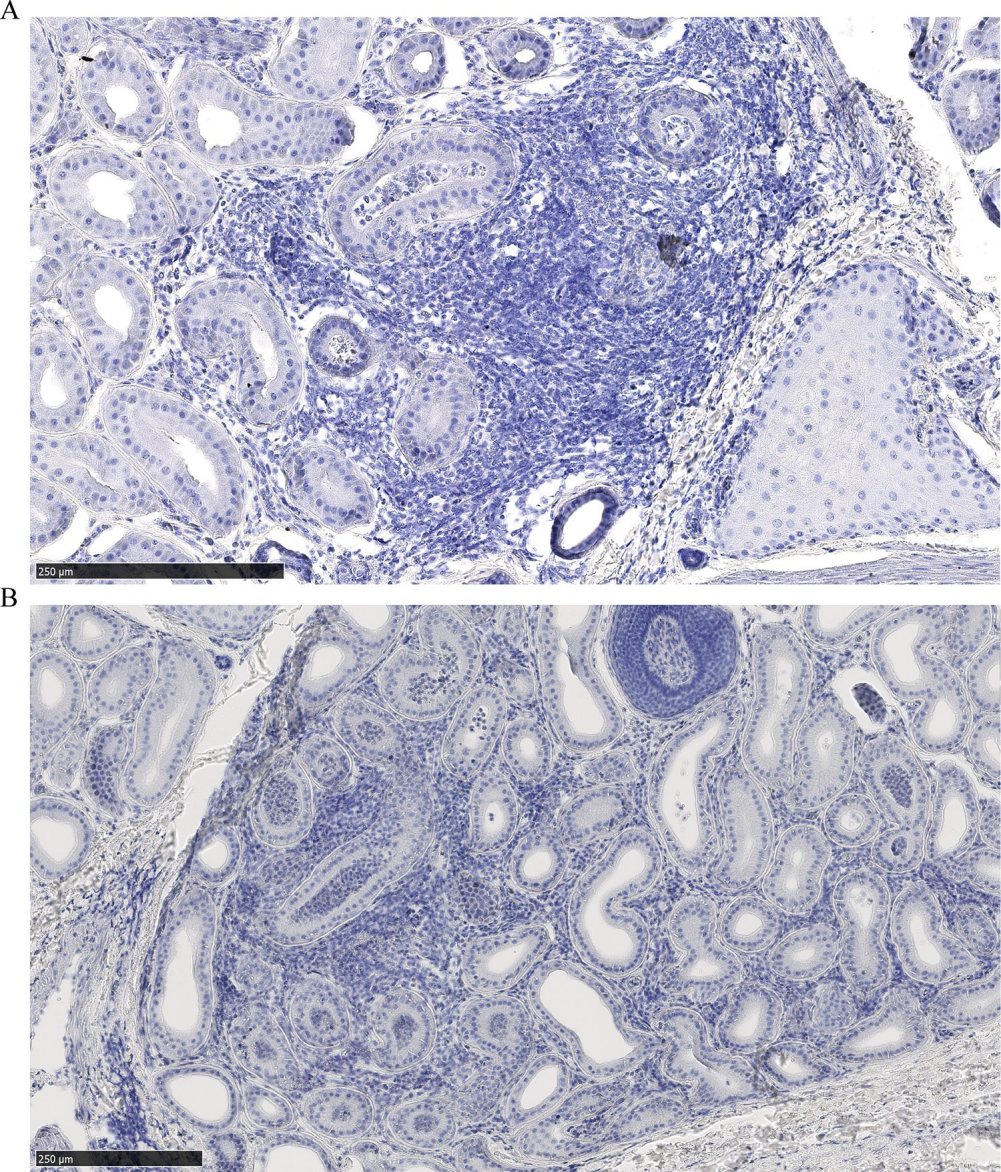
Leukocytic infiltration of sudoriferous gland lumen. (A) Female mule deer hind leg interdigital glands of with PrP^CWD^ deposits in other regions of the section, and (B) an uninfected male mule deer.

Dense leukocytic infiltrates unrelated to CWD were observed within all 2017 mule deer interdigital gland samples examined by histology—consistent with past observations [26, 27]. No PrP^CWD^ was observed in the interdigital glands of CWD-negative deer (7 mule deer and 2 white-tailed deer). Histology was available for two CWD-infected mule deer collected in 2017. PrP^CWD^ was observed in the interdigital gland of a CWD-infected female mule deer, but not in a CWD-infected male mule deer. Of note, the male had visibly less severe leukocytic infiltration than the female. The CWD-infected female mule deer presented with PrP^CWD^ among leukocytic infiltrates between and adjacent to sudoriferous (Figs 2 and 3) and sebaceous glands (Fig 3B). Further PrP^CWD^ immunolabeling was identified within the acrosyringeal epidermis of a dilated, blocked sudoriferous duct (miliaria rubra) (Fig 4) [45, 46]. Deposition of PrP^CWD^ was confirmed by analysis of sequential tissues sections (S2 and S3 Figs in S1 File). Acrosyringium structure was confirmed by serial hematoxylin and eosin sections (S4 Fig in S1 File). PrP^CWD^ was located close to the epidermis with 6 foci of PrP^CWD^ immunolabeling averaging 623μm from the external surface of the epidermal stratum granulosum. Aberrant infiltration of sudoriferous tubule lumen by leukocytes was observed in uninfected and CWD-infected mule deer without PrP^CWD^ (Fig 5, S4 Fig in S1 File). PrP^CWD^ was not observed in nerves associated with interdigital glands.

### Detection of interdigital PrP^CWD^ by RT-QuIC and sPMCA

Interdigital glands of male mule deer forefeet harvested in 2018 on CFB Wainwright, Alberta were tested for the presence of PrP^CWD^ by sPMCA in a blinded study. 19 interdigital glands from the forefeet of 10 male mule deer were tested (only one interdigital gland was collected from one of the animals). Seven rounds of sPMCA were required to amplify detectable levels of PrP^CWD^. All samples were retested by sPMCA in a second trial to examine result consistency (S5 Fig in S1 File). sPMCA yielded several inconsistencies between trials and a high rate of false positives when provided the provincial CWD surveillance results (S1 Table in S1 File). sPMCA successfully identified 2 of 3 CWD-infected animals, but with 3 false positives from a sample size of 10 animals.

**Fig 6:**
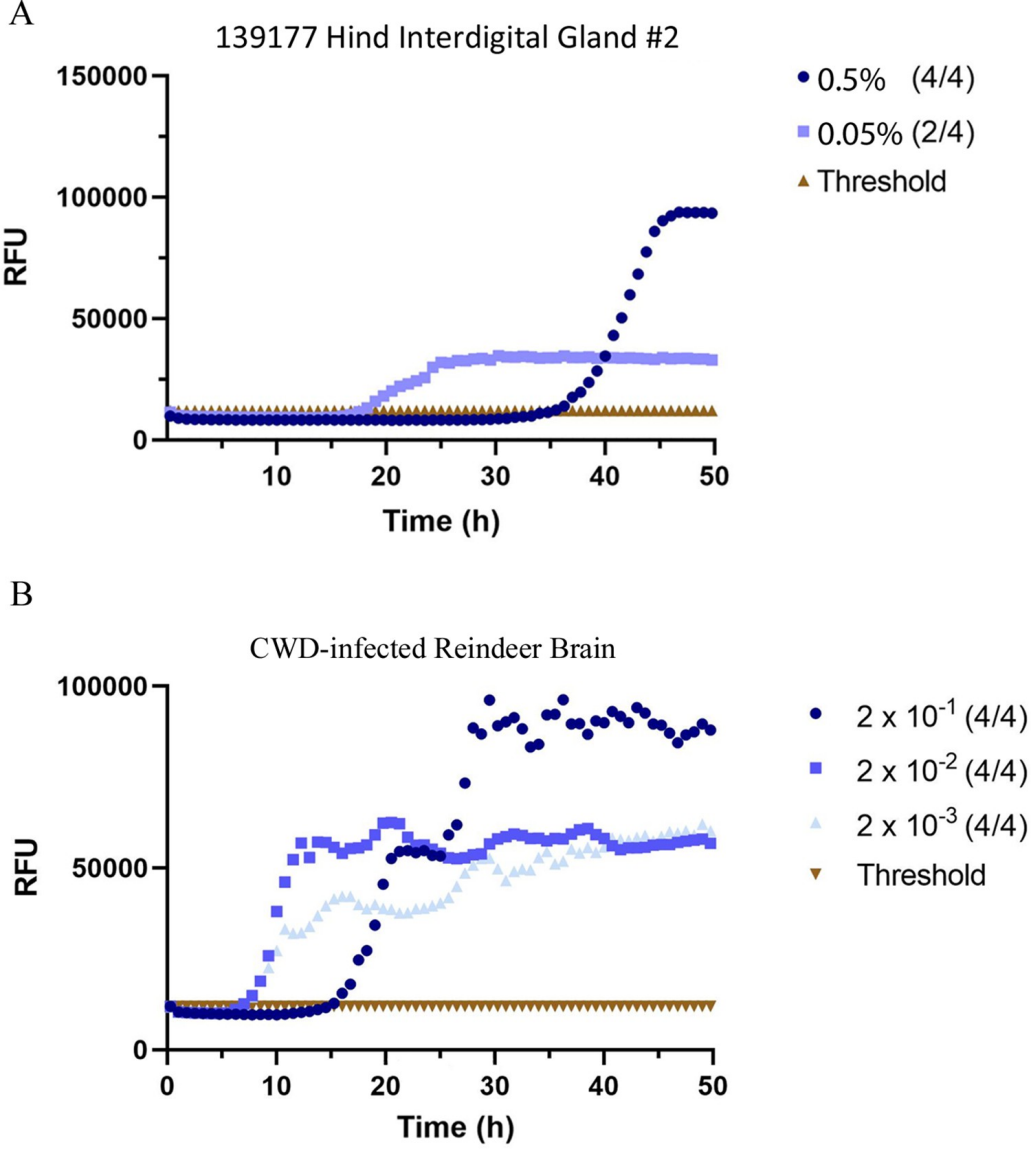
RT-QuIC detection of PrP^CWD^ in blinded mule deer leg interdigital glands. (A) Representative example of single-round RT-QuIC detection of PrP^CWD^ in one interdigital gland of a CWD-infected mule deer. (B) CWD-infected reindeer brain homogenate positive control. Samples were tested in quadruplicate with average relative fluorescence units (RFU) displayed. Gland homogenates were diluted to 0.5% and 0.05% (w/v) final concentrations.

The RT-QuIC analysis included 4 interdigital, 2 tarsal, and 2 metatarsal glands of a clinically-positive female mule deer and 19 interdigital glands from 10 male mule deer also used for sPMCA testing. Blinded RT-QuIC testing returned faster, single-round results with accurate diagnostic results (Fig 6, Table 1). The best results were obtained using 0.5% (w/v) interdigital gland homogenates. RT-QuIC of interdigital glands correctly identified all CWD-positive animals with no false positives or negatives (Table 1, S6 Fig in S1 File). Infectivity was not identified by RT-QuIC in the metatarsal and tarsal glands of the clinically-affected deer. Positive testing 0.5% interdigital gland samples crossed the negative control threshold between 30 and 46 hours—later than all CWD-infected reindeer brain positive controls. All CWD-infected mule deer were determined to have wildtype *PRNP* protein sequences with the exception of individual 136227 which was heterozygous for a previously known D20G polymorphism [47].

**Table 1:** Blinded detection of PrP^CWD^ in interdigital (ID), metatarsal (MET), and tarsal (TAR) glands by RT-QuIC.

Deer Tissue &	Provincial CWD	RT-QuIC				
Identification	Status	0.5% GH			0.05% GH	
136179 ID	Negative	n = 2	(0/4)	(0/4)	(0/4)	(0/4)
136180 ID	Negative	n = 2	(0/4)	(0/4)	(0/4)	(0/4)
136207 ID	Negative	n = 2	(0/4)	(0/4)	(0/4)	(0/4)
136214 ID	Negative	n = 2	(0/4)	(0/4)	(0/4)	(0/4)
136216 ID	Negative	n = 2	(0/4)	(0/4)	(0/4)	(0/4)
136221 ID	Negative	n = 2	(0/4)	(0/4)	(0/4)	(0/4)
136227 ID	**Positive**	n = 2	(1/4)	**(2/4)**	(0/4)	(1/4)
136229 ID	Negative	n = 2	(0/4)	(0/4)	(0/4)	(0/4)
136230 ID	**Positive**	n = 2	(1/4)	**(4/4)**	(0/4)	**(2/4)**
136237 ID	**Positive**	n = 1	**(2/4)**		(1/4)	
139177^a^	**Positive**	n = 4	**(3/4)**	**(4/4)**	(1/4)	**(2/4)**
ID			**(4/4)**	**(4/4)**	(0/4)	(0/4)
MET		n = 2	(1/4)	(0/4)	(0/4)	(0/4)
TAR		n = 2	(0/4)	(0/4)	(0/4)	(0/4)

Gland homogenates (GH) (0.5% or 0.05% (w/v)) were tested by RT-QuIC in quadruplicate.

Total number of glands tested (n) are shown along with the assay results.

Threshold for positivity is 5 standard deviations above average negative controls. Samples with 2 or more positive replicates are bolded.

^a^Individual 139177 was a mule deer that was clinically affected with CWD.

### sPMCA detection of PrP^CWD^ in soil trapped in the hoof of a CWD-infected mule deer

**Fig 7:**
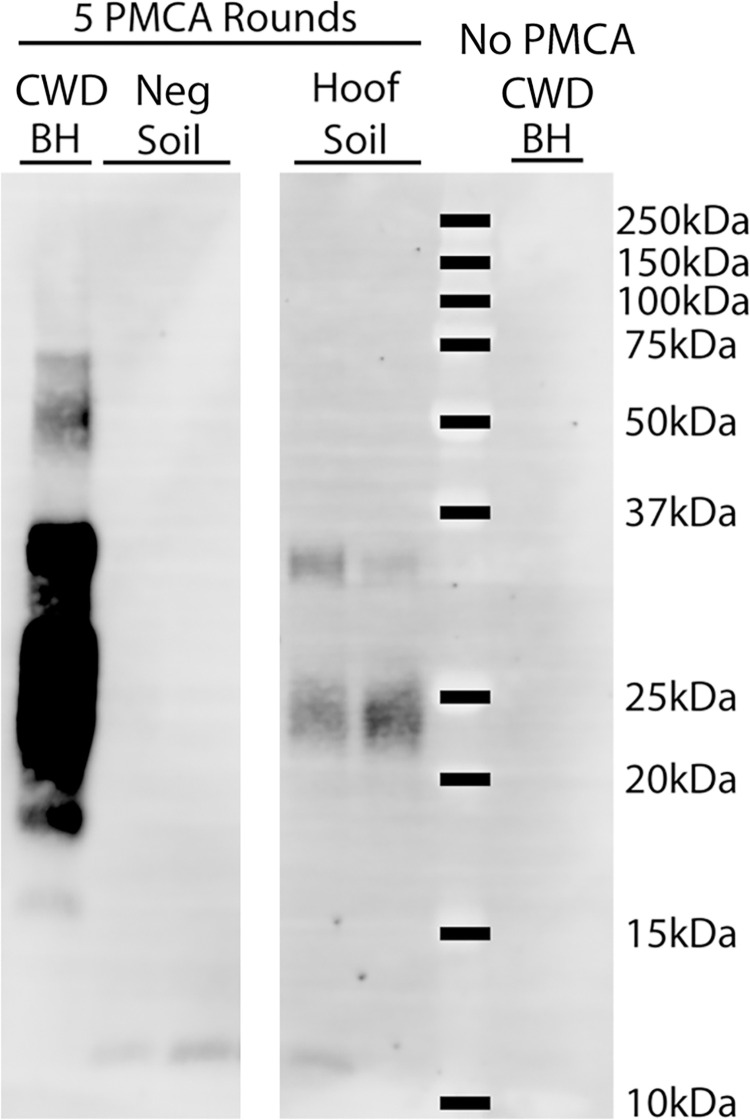
Detection of PrP^CWD^ from soil found between the hoof digits of a CWD-infected mule deer. Soil was assayed for the presence of PrP^CWD^ seeding ability by sPMCA. Samples assayed included soil obtained from between the hoof digits of CWD clinically-positive female mule deer 139177 and soil from a CWD-free region (Neg soil). Following a PrP^CWD^-extraction procedure, samples were subjected to 5 rounds of PMCA in duplicate. Samples were compared to 0.1% CWD-infected brain homogenate (BH) controls with and without PMCA.

A soil sample was obtained from between the hoof digits of clinically positive mule deer 139177. All four interdigital glands of this deer tested positive by RT-QuIC, but fixed samples were not available for immunohistochemistry. CWD-infected brain, the hoof soil sample, and negative control soil from Alberta were analyzed by 5 rounds of sPMCA. The hoof soil amplified proteinase K-resistant material—indicating the possible presence of PrP^CWD^ (**Fig 7**).

## Discussion

PrP^CWD^ was detected in the interdigital glands of CWD-positive mule deer. CWD-infected white-tailed deer hooves were not available for this study. PrP^CWD^ accumulation of the female mule deer occurred primarily in the immune cells of the glandular leukocytic infiltrates (Figs 2 and 3). A prior survey of PrP^C^ in the interdigital glands identified PrP^C^ expression in the structures associated with the PrP^CWD^ we describe—namely the infiltrating immune cells, but also the sudoriferous and sebaceous glands [26]. CWD infection initiation and establishment in the interdigital gland is possible; however, centrifugal spread of PrP^CWD^ to the interdigital glands is a strong and more plausible possibility. The presence of PrP^CWD^ colocalizing with infiltrating leukocytes is indicative of centrifugal spread by such cells. Circulating immune cells carrying PrP^CWD^ or PrP^Sc^ in prion infected animals are known to be concentrated in sites of inflammation—making them easier to identify by immunohistochemistry [48–54]. The interdigital glands of mule deer possess numerous dense leukocytic infiltrates, indicating that they are subject to more inflammation relative to other integumentary glands or deer [26, 27, 55] which likely explains why infectivity was readily observed in the interdigital gland and not in the metatarsal or tarsal glands (Table 1). Leukocytic infiltrates in the interdigital glands of white-tailed deer are less dense or are not noted entirely relative to mule deer [26–28]. The reduced propensity for white-tailed deer to have dense leukocytic infiltrates in the interdigital glands may translate into less sensitive detection of PrP^CWD^ using interdigital gland tissues for that species. The presence of leukocytic infiltrates in the interdigital skin of healthy sheep [56, 57] indicate that interdigital tissue testing could also be valuable for diagnosis of scrapie.

We report the presence of PrP^CWD^ by immunohistochemistry within the interdigital gland of a CWD-infected female mule deer. The other CWD-infected mule deer examined by immunohistochemistry had no observable PrP^CWD^ which could be attributed to lesser grade of immune cell infiltration in that individual. Cautioned interpretation of the results is warranted when considering that PrP^CWD^ was observed by immunohistochemistry in only one of two CWD-infected animals where fixed samples were available. Immunolabeling of PrP^CWD^ was observed immediately adjacent to sudoriferous and sebaceous glands (Fig 3A and 3B). The source of the PrP^CWD^ observed within the acrosyringeal epidermis (Fig 4B) could either represent a novel prion tropism or, based on the dense lymphocytic infiltration, PrP^CWD^-containing lymphocytes infiltrating the epidermis. The PrP^CWD^ immunolabeling was located closer to the lumen of the acrosyringium and was not observed among the adjacent infiltrating interstitial lymphocytes (Fig 4B, S3 Fig in S1 File). The novel tropism of the acrosyringium is supported by PrP^C^ expression being previously observed in the interdigital gland epidermis including that of glandular ducts [26] but is countered by higher cell division rates which are generally inversely correlated with cell propensity for prion replication [58]. Prions have been detected in the skin of a variety of prion diseases [59–63]. Using proteinase K-digested paraffin embedded tissue blots, Thomzig *et al*., identified structures with PrP^Sc^ deposition in terminal stage hamsters infected with the 263K prion strain [60]. Differing from our findings, the authors found PrP^Sc^ in a variety of cutaneous nerve fibers, plexuses, and follicular hair innervations.

Dissemination of CWD prions into the environment from the interdigital glands is a possible consequence of infectivity in a secreting gland. Cervid salivary glands have long been known to be a source of PrP^CWD^ [5–11, 64–66], so finding PrP^CWD^ in other secretory glands could be expected. Direct secretion of PrP^CWD^ could occur as infiltrating immune cells disrupt the architecture of the sebaceous glands (Fig 3B) or invade the sudoriferous tubules (Fig 5, S4 Fig in S1 File). The presence of PrP^CWD^ immunolabeling within the acrosyringeal epithelium is supportive of possible interdigital gland secretion. An analogous mechanism of integumentary gland secretion of prions exists in scrapie. Comparable to our results, immunohistochemistry of sheep with mastitis identified scrapie-associated PrP^Sc^ in lymphoid follicles adjacent to mammary gland ducts and within mammary gland ducts and acini [52–54, 67]. Mastitis is not required for secretion of PrP^Sc^ into milk of scrapie-infected ewes [52, 53, 68]. More disseminated leukocytic infiltration in the interdigital and mammary glands is expected to assist with visualizing PrP^CWD^ and PrP^Sc^ by immunohistochemistry. Visibly less immune cell infiltration of the interdigital glands of the infected male mule deer with available histology may explain why PrP^CWD^ was not visualized by immunohistochemistry in those samples.

Detection of PrP^CWD^ from soil adventitiously lodged between the hoof digits of a clinically-affected deer (Fig 7) that had all four interdigital glands test positive by RT-QuIC lends credence to the theory of soil contamination by interdigital gland secretions. Due to the avidity of binding of prions to soil and soil components, detection using sPMCA is challenging. Analysis of additional soil collected from deer hooves will validate these current results and provide further information regarding the amount of infectivity present. The feasibility of uninfected deer being exposed to and infected by PrP^CWD^ through the interdigital glands could not be determined by our study. Some insight can be provided by the lack of observed PrP^CWD^ by immunohistochemistry in 9 uninfected deer, and was not detected by RT-QuIC in 7 other uninfected deer.

Our study demonstrated that RT-QuIC provides more specific and sensitive interdigital gland prion detection than sPMCA under the conditions tested. Slow interdigital gland sample RT-QuIC reactions (0.5% homogenates crossing the negative control thresholds between 30 and 46 hours) indicate that the seeding activity in the tissue homogenate is very low, or that reaction conditions for gland tissue could be optimized. Inaccurate detection of PrP^CWD^ in the interdigital glands of deer by seven rounds of sPMCA is further suggestive of low infectivity. False positive results can be attributed to the high number of PMCA rounds [40]. It is possible that unidentified components of the interdigital gland homogenates, possibly ceraceous gland secreta, inhibit the PMCA reactions. Disintegration of sebum and other homogenate components by sonication may sequester prion seeding activity. The RT-QuIC methodology can be optimized by using recPrP substrates of species other than mouse. For the RT-QuIC reactions, uninfected gland homogenates may have been more appropriate negative controls but, as our samples were blinded, we did not have material for this use. An opportunity exists to explore post- and antemortem CWD diagnosis using interdigital gland biopsies or possibly the hair and secretions. Larger sample sizes of both white-tailed deer and mule deer will be required to determine what stage of disease PrP^CWD^ first accumulates in the interdigital glands and for determining the diagnostic value of testing interdigital gland tissues. The small sample size we report prevents us from determining the frequency and abundance of PrP^CWD^ accumulation in interdigital glands. More importantly, any possible contribution of interdigital gland PrP^CWD^ secretion into the environment and any associated disease transmission effects has yet to be investigated.

Detection of PrP^CWD^ in the interdigital glands is a novel finding that has implications for ante- and post-mortem prion diagnosis. RT-QuIC was determined to be the preferred method of prion detection over sPMCA using interdigital gland homogenates. The presence of PrP^CWD^ in the interdigital glands is likely reflective of the dense leukocytic infiltrates that are commonly observed in the interdigital glands of mule deer. Future bioassays will assess the infectivity present in the interdigital glands relative to other tissues. Secretion of PrP^CWD^ into the environment is suspected based on immunohistochemical identification of PrP^CWD^ near secreting sebaceous and sudoriferous glands. Identification of PrP^CWD^ in additional interdigital glands would suggest a role for these glandular secretions in horizontal CWD transmission. Exposure of uninfected animals through the interdigital glands is possible but is currently lacking evidence when considering that PrP^CWD^ was only detected in the interdigital glands of animals determined to be infected with CWD by existing CWD-surveillance methods.

## Supporting information

S1 Raw images (PDF)

S1 File (PDF)
