# Supplementary figures and images for: Chronic wasting disease prions in mule deer interdigital glands
Source: PLoS One. 2022 Oct 3;17(10):e0275375. doi: 10.1371/journal.pone.0275375 (PMC9529147; doi:10.1371/journal.pone.0275375)

S1 raw images of Figure 6.

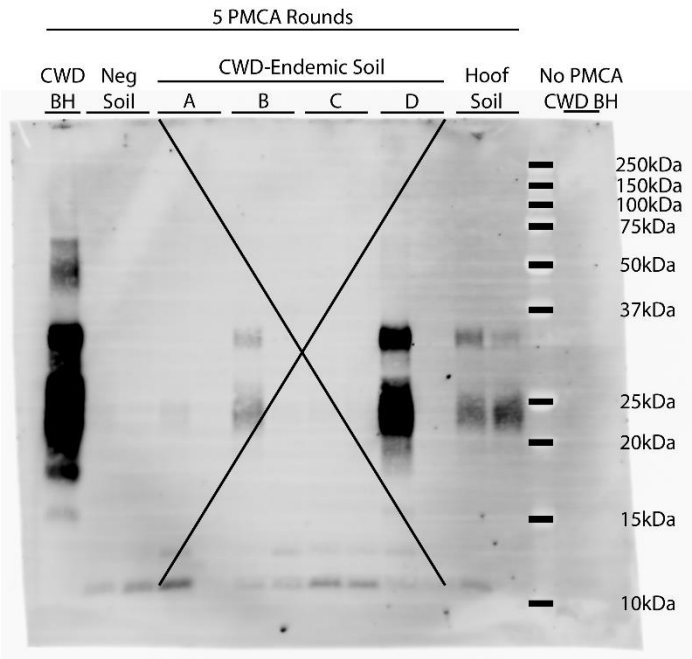

Supplement: S1 Raw images — (PDF) [file pone.0275375.s001.pdf]
