# Supplementary material for: Chronic wasting disease prions in mule deer interdigital glands
Source: PLoS One. 2022 Oct 3;17(10):e0275375. doi: 10.1371/journal.pone.0275375 (PMC9529147; doi:10.1371/journal.pone.0275375)
Supplement: S1 File — (PDF) [file pone.0275375.s002.pdf]

A

B

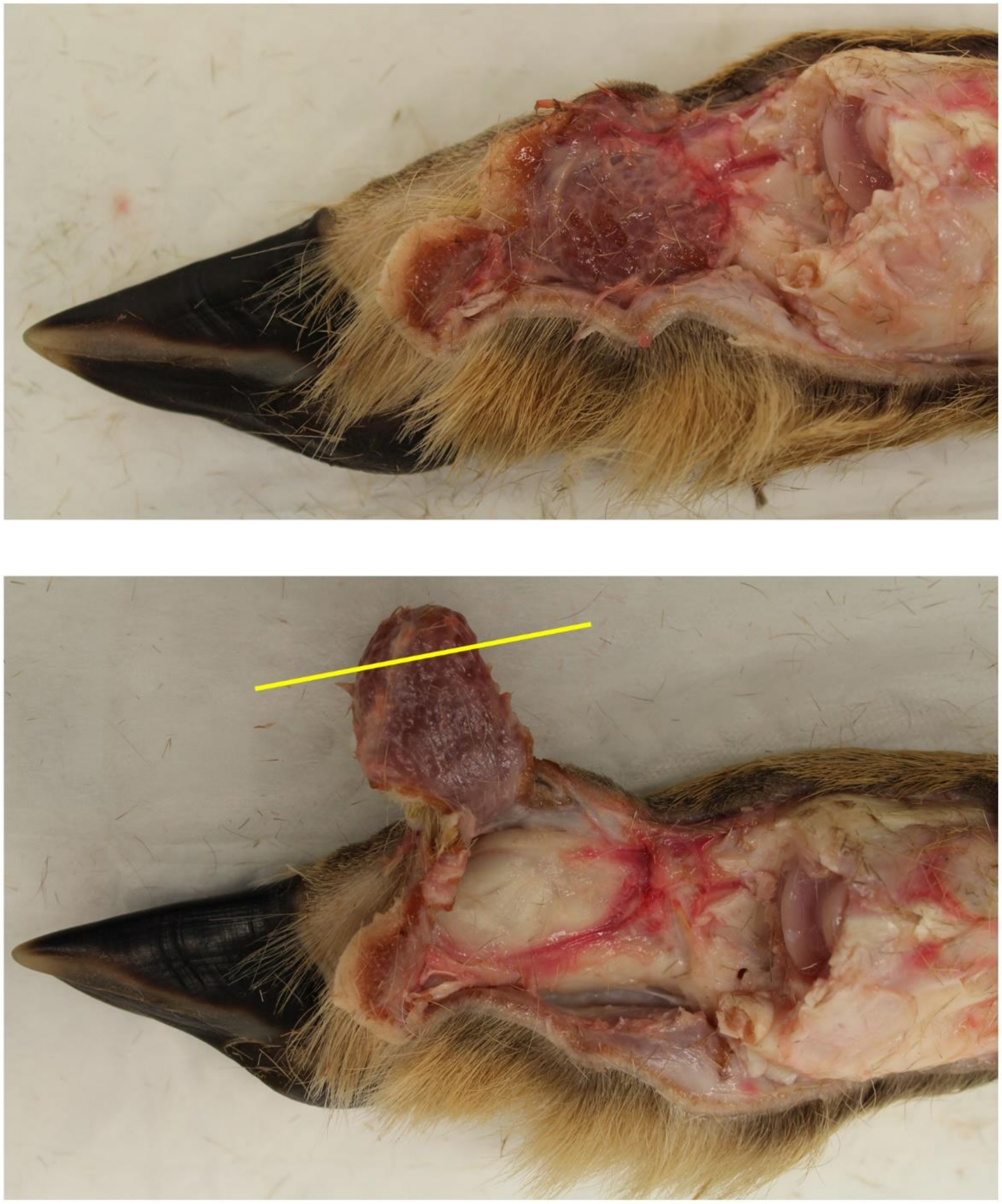

S1 Fig. Extraction of the interdigital gland fundus for homogenization. (A) Half of the hoof was disarticulated leaving the entire interdigital gland intact intact. (B) The glandular sac is exposed and the fundus excised by cutting at the yellow line.

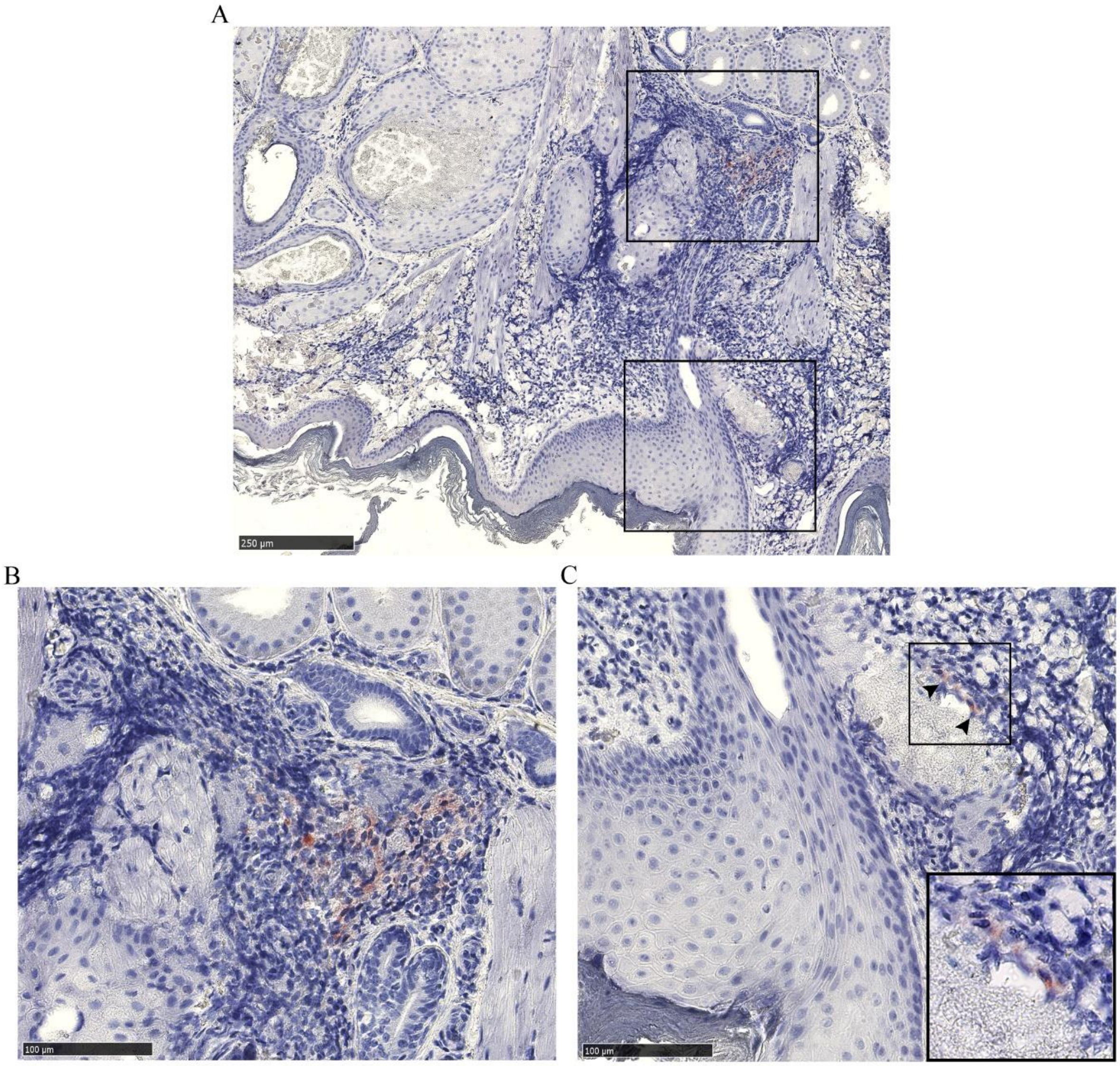

S2 Fig. Adjacent section to Fig 3 showing interdigital gland PrP^{CWD} immunolabeling in a CWD-infected mule deer. (A) Immune cell infiltrates near the epidermis, between sebaceous and sudoriferous glands. (B) Increased magnification of the upper inset showing PrP^{CWD} immunolabeling adjacent to sudoriferous gland tubules. (C) Increased magnification of the lower inset showing PrP^{CWD} immunolabeling (red, arrows) adjacent to a sebaceous glandular element. Inset shows PrP^{CWD} with increased magnification. PrP^{CWD} was immunolabeled with anti-PrP BAR224 (1:2,000).

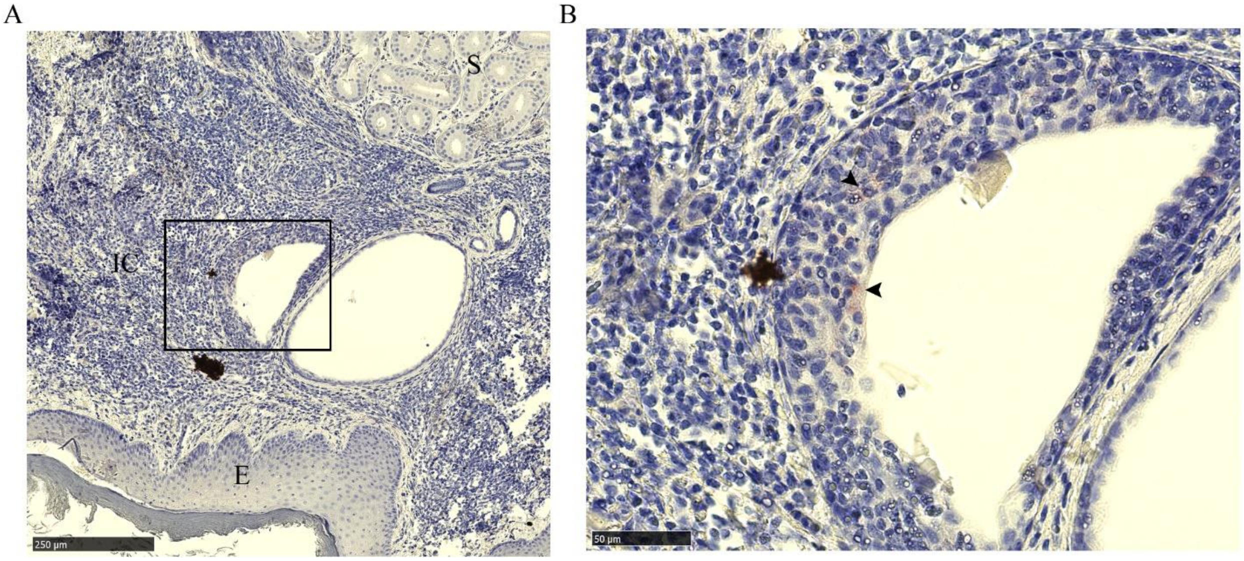

S3 Fig. Sequential section to Fig 4 showing hind interdigital gland PrP^{CWD} immunolabeling in the acrosyringeal epidermis of a dilated sudoriferous gland from a CWD-infected mule deer. (A) Immune cell infiltrates and vessels between the epidermis and sudoriferous glands. (B) Increased magnification of the inset showing acrosyringeal epidermis with PrP^{CWD} (arrows). PrP^{CWD} was immunolabeled with anti-PrP BAR224 (1:2,000). Abbreviations: E, epidermis; IC, immune cell infiltrates; S, sudoriferous glands.

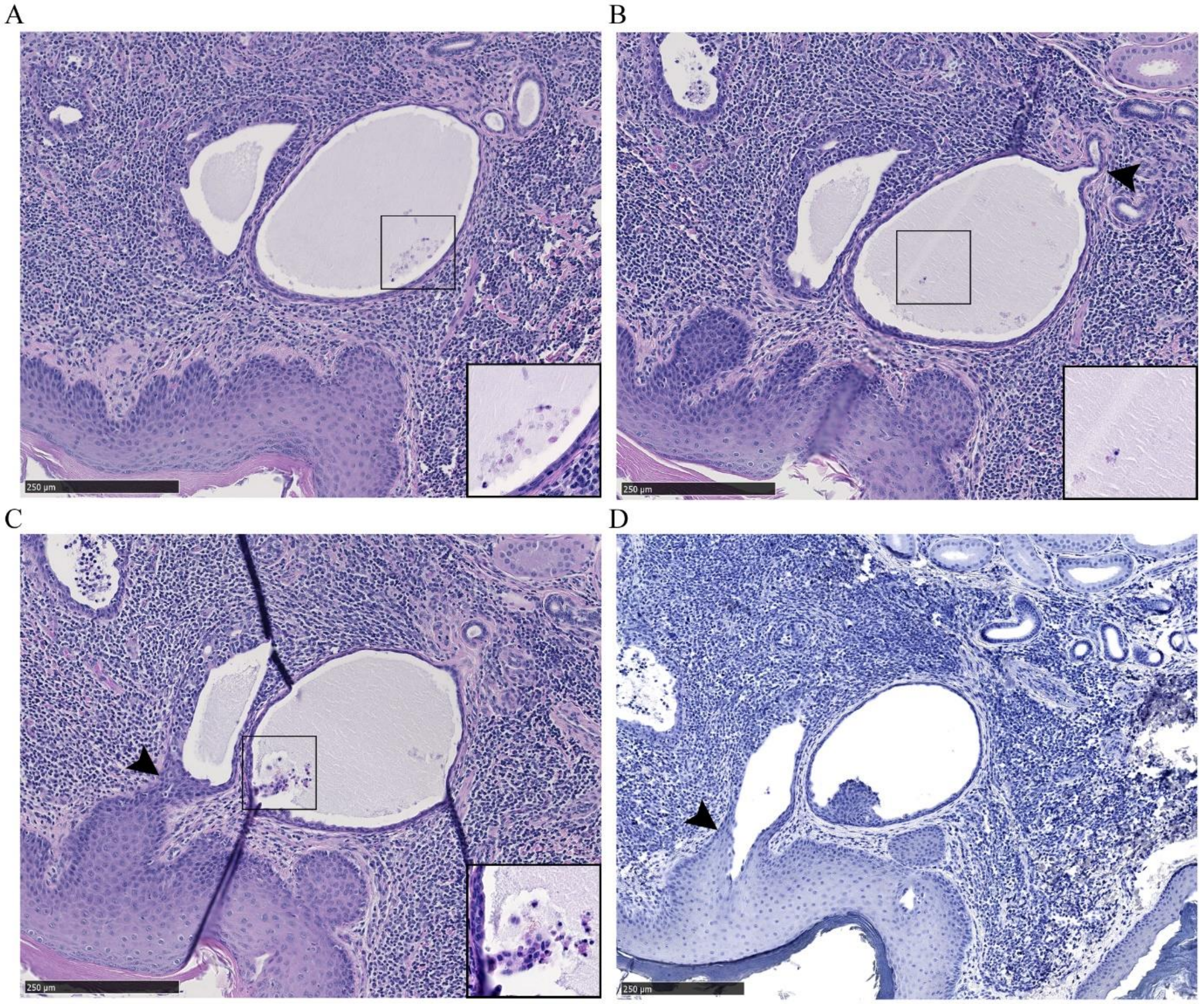

S4 Fig. Identification of the acrosyringium and miliaria. A) Section adjacent to Figure 4-4A which presented with PrP^{CWD} immunolabeling in the acrosyringeal epidermis. (B) Deeper sections showing the joining of the larger dilated sudoriferous duct to narrower coils (black arrow) indicating miliaria profundum. (C-D) Visualization of the dilated acrosyringeal epidermis (miliaria rubra) joining to the external epidermis (black arrows). Ectopic lymphocytes in the lumen of the larger dilated tubule are observed in most sections (insets). Sections (A-C) were stained by haematoxylin and eosin. Section (D) was probed with BAR224 (1:2,000) with haematoxylin counterstaining.

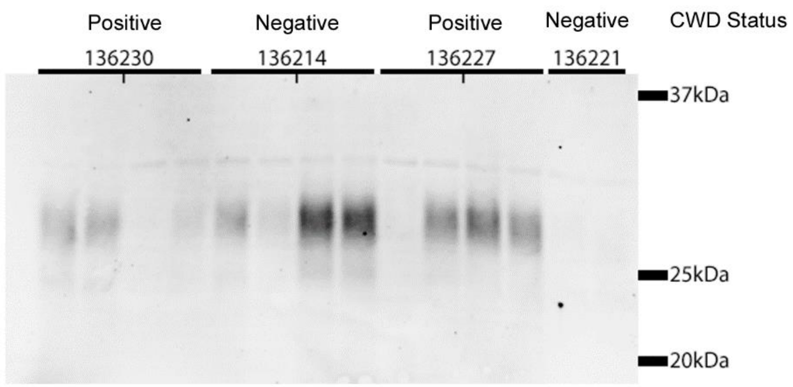

S5 Fig. Detection of PrP^{CWD} in blinded mule deer leg interdigital gland samples by sPMCA. Representative western blot of 7th round sPMCA testing of each interdigital gland in duplicate.

S6 Fig. Blinded RT-QuIC analysis of leg glands.

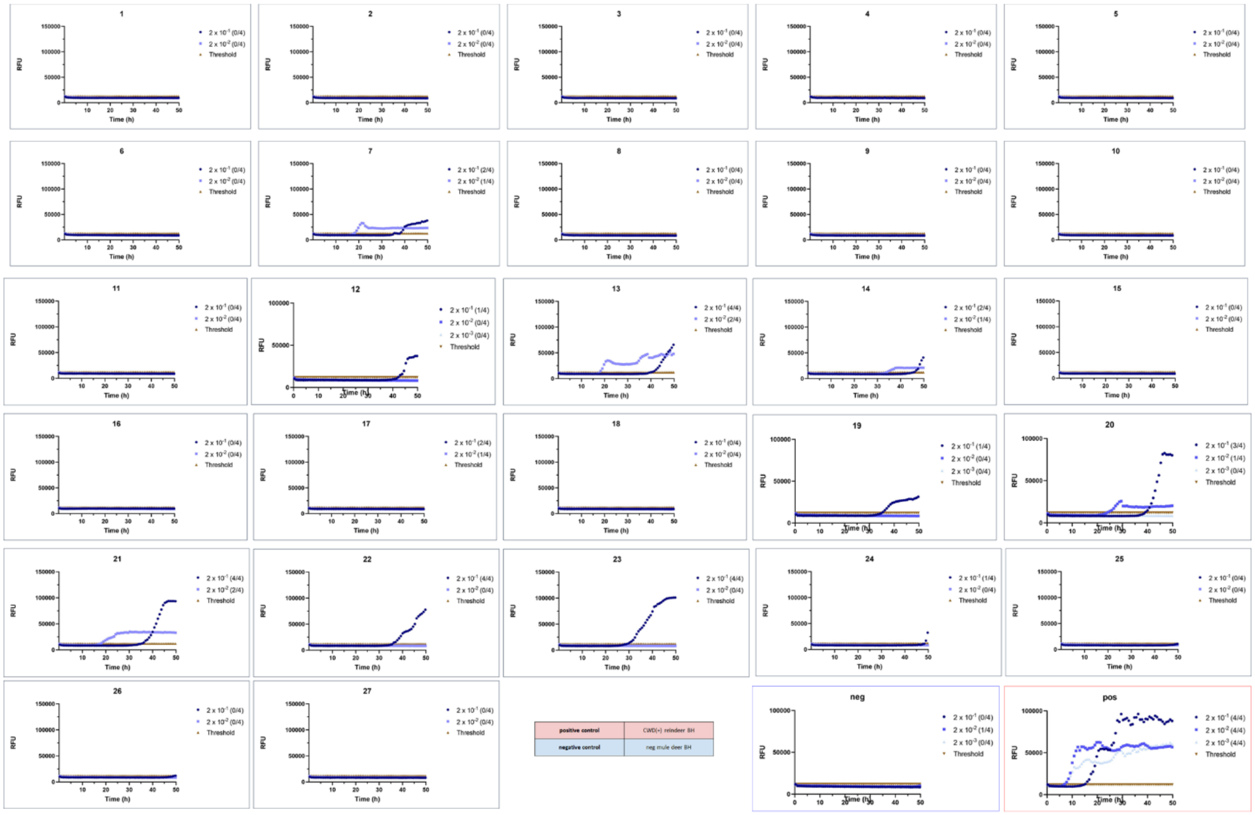

Blinded RT-QuIC Samples	0.50%	0.05%
#1 136214B;	0/4	0/4
#2 136229B;	0/4	0/4
#3 136221B;	0/4	0/4
#4 136207B;	0/4	0/4
#5 136180A;	0/4	0/4
#6 136221A;	0/4	0/4
#7 136227B;	2/4	1/4
#8 136216A;	0/4	0/4
#9 136229A;	0/4	0/4
#10 136179B;	0/4	0/4
#11 136216B;	0/4	0/4
#12 136227A;	1/4	0/4
#13 136230B;	4/4	2/4
#14 136237A;	2/4	1/4
#15 136207A;	0/4	0/4
#16 136214A;	0/4	0/4
#17 136179A;	0/4	0/4
#18 136180B;	0/4	0/4
#19 136230A	1/4	0/4
#20 139177 Fore Interdigital leg 1	3/4	1/4
#21 139177 Fore Interdigital leg 2	4/4	2/4
#22 139177 Hind Interdigital leg 1	4/4	0/4
#23 139177 Hind Interdigital leg 2	4/4	0/4
#24 139177 Hind Metatarsal leg 1	1/4	0/4
#25 139177 Hind Metatarsal leg 2	0/4	0/4
#26 139177 Hind Tarsal leg 1	0/4	0/4
#27 139177 Hind Tarsal leg 2	0/4	0/4

S1 Table 1. Blinded detection of PrP^{CWD} in interdigital (ID), metatarsal (MET), and tarsal (TAR) glands by sPMCA and RT-QuIC.

Deer Tissue & Identification	CWD Status	sPMCA		RT-QuIC	
		Trial 1	Trial 2	0.5% GH	0.05% GH
136179 ID	Negative	n=2 (0/4)	(0/4)	n=2 (0/8)	(0/8)
136180 ID	Negative	**n=2 (2/4)**	**(2/4)**	n=2 (0/8)	(0/8)
136207 ID	Negative	n=2 (0/4)	(1/4)	n=2 (0/8)	(0/8)
136214 ID	Negative	n=2 (1/4)	**(3/4)**	n=2 (0/8)	(0/8)
136216 ID	Negative	n=2 (0/4)	(0/4)	n=2 (0/8)	(0/8)
136221 ID	Negative	**n=2 (2/4)**	(1/4)	n=2 (0/8)	(0/8)
136227 ID	**Positive**	**n=2 (4/4)**	**(3/4)**	**n=2 (3/8)**	(1/8)
136229 ID	Negative	n=2 (0/4)	(0/4)	n=2 (0/8)	(0/8)
136230 ID	**Positive**	**n=2 (3/4)**	**(3/4)**	**n=2 (5/8)**	**(2/8)**
136237 ID	**Positive**	n=1 (0/2)	(0/2)	**n=1 (2/4)**	(1/4)
139177[a] ID				**n=4 (15/16)**	**(3/16)**
MET	**Positive**			n=2 (1/8)	(0/8)
TAR				n=2 (0/8)	(0/8)

Number of glands (n) tested by 7 rounds of sPMCA in duplicate or by one round of RT-QuIC in quadruplicate.
Gland homogenates (GH) (0.5% or 0.05% (w/v)) were tested by RT-QuIC in quadruplicate.
[a]Individual 139177 was a mule deer clinically affected with CWD.
